# Structural, Crystallographic, and Thermal Properties of Bi_12_GeO_20_ for Technological Applications

**DOI:** 10.1021/acsomega.4c09220

**Published:** 2025-06-12

**Authors:** Gozde Altuntas, Mehmet Isik, Nizami Mamed Gasanly

**Affiliations:** † Faculty of Technology, Department of Metallurgical and Materials Engineering, 37511 Gazi University, 06560 Ankara, Türkiye; ‡ Department of Biomedical Engineering, Faculty of Engineering and Architecture, 510312 İzmir Bakırçay University, 35665 Izmir, Türkiye; § Biomedical Technologies Design Application and Research Center, İzmir Bakırçay University, 35665 Izmir, Türkiye; ∥ Department of Physics, Middle East Technical University, 06800 Ankara, Türkiye; ⊥ Virtual International Scientific Research Centre, Baku State University, 1148 Baku, Azerbaijan

## Abstract

In this work, we present the structural and thermal properties of the Bi_12_GeO_20_ crystal grown using the Czochralski method. X-ray diffraction (XRD) analysis elucidated the crystal structure, while scanning electron microscopy (SEM) and energy-dispersive spectroscopy (EDS) provided information on morphology and elemental composition, respectively. Many peaks belonging to the cubic crystalline structure were observed in the XRD pattern. Thermal behavior was extensively investigated using thermal gravimetric analysis (TGA) and differential scanning calorimetry (DSC). In the crystal, where no serious weight loss was observed up to 863 K, two different weight loss behaviors were observed in the regions between 863 and 1048 K and 1048–1193 K. The behavior observed in these two different regions was associated with two different decomposition and/or evaporation processes, and the activation energies of these processes were determined using the Coats–Redfern equation as 99.7 and 33.1 kJ/mol. As a result of DSC measurements performed with different heating rates, it was observed that an endothermic process occurred around 618 K. The activation energy of this process was found to be 495 kJ/mol using the Kissenger equation. Our findings reveal details about the crystal’s structural arrangement, morphological property, and decomposition/phase transitions, shedding light on the crystal’s potential technological applications in various fields.

## Graphical abstract

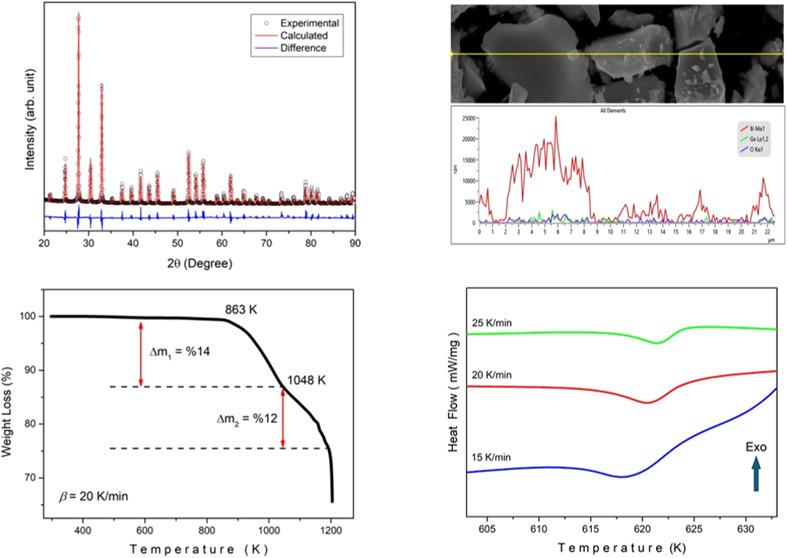

## 1 Introduction

Bi_12_MO_20_ (M:Ge,Si,Ti) compounds, commonly referred to as sillenites, are well-known for their unique properties, making them highly attractive for diverse technological applications, including photocatalysis, electro-optics, optical data processing, and ferroelectricity.^1–4^ Bismuth-based compounds, which are a component of this family, have low toxicity and radioactivity, making them suitable for a variety of applications. These compounds demonstrate notable photocatalytic activity, primarily attributed to their effective absorption and utilization of visible light.^5^ Among these compounds, bismuth germanium oxide (Bi_12_GeO_20_), commonly termed BGO, is a particularly significant member of the sillenite group. It possesses a range of favorable properties, including photocatalytic ability, photoconductivity, photorefraction, and piezoelectricity.^6,7^ BGO has attracted much attention in numerous research papers due to its potential applications in holographic recording, optical information processing, acoustic wave propagation, and nonlinear optical devices.^8–10^ Moreover, its significant magneto-optical quality designates it as a promising material for Faraday rotator crystals, imparting potential in sensor systems.^11^

Bi_12_GeO_20_ exhibits a cubic *I*2̅ 3̅ space group crystalline structure, with a reported lattice constant of *a* = 1.0304 nm.^12^ As shown in Figure 1, GeO_4_ tetrahedra occupy the corners of the cube, while Bi atoms are centrally positioned in the cube, each surrounded by oxygen atoms. Regarding its optical properties, the band-gap energy of BGO is conventionally acknowledged to be around 3.2 eV.^13^ However, alternative studies have reported significantly lower band-gap energies between 2.3 and 2.9 eV.^14–16^ These variations in reported band-gap energies have been attributed to the presence of defect centers within the crystal structure. Sillenite compounds are known to possess a notable number of defects, which introduce energy levels within the forbidden band gap. These defect-induced energy levels play a significant role in absorption processes, contributing to the observed variations in band-gap energy. Although the presence of these defect centers is considered negative, it adds positive effects to the material for visible optoelectronic applications.^9,17^

**Figure 1:**
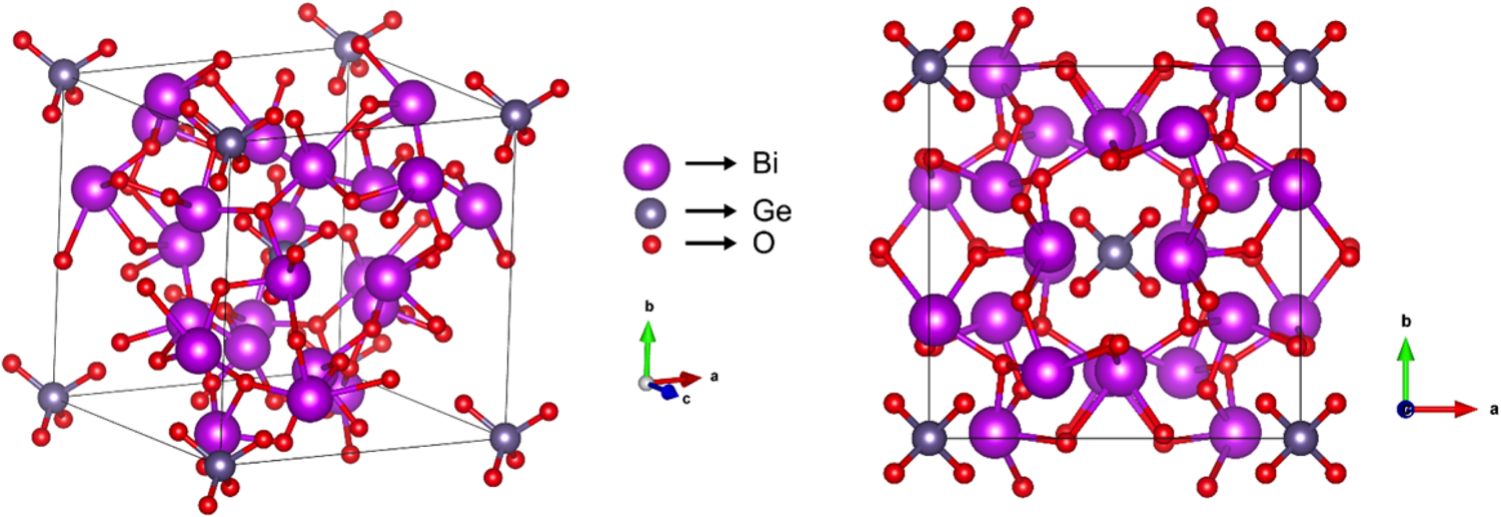
Crystal structure of the Bi_12_GeO_20_ sillenite.

Studies on BGO material so far have largely been about understanding the structural and optical properties of the material. Studies on understanding the thermal properties of BGO are limited and are generally at a theoretical level. Temperature-dependent heat capacity and thermal expansion coefficient were determined theoretically using the quasi-harmonic Debye model.^18^ Experimentally, the temperature-dependent heat capacity, thermal conductivity, and thermal diffusivity of the BGO compound were previously reported.^19^ Determining the thermal properties of BGO is of great importance, as it directly affects its performance and applicability in various fields. Understanding the thermal behavior of BGO through techniques such as thermogravimetric analysis (TGA) and differential scanning calorimetry (DSC) is crucial for optimizing its processing conditions, enhancing its stability, and elucidating its potential in thermal management applications. Therefore, this article aims to comprehensively explore the thermal properties of the Bi_12_GeO_20_ crystal for the first time through detailed TGA and DSC analyses, shedding light on its thermal stability, phase transitions, and decomposition behavior.

## 2 Experimental Details

Bi_12_GeO_20_ crystals were grown by using the Czochralski method. A precise mixture of Bi_2_O_3_ and GeO_2_ in a 6:1 molar ratio was melted in a cylindrical crucible. Subsequently, a rotating seed crystal was immersed in the molten material and slowly withdrawn from the crucible, allowing for a controlled crystallization. The photograph of the grown crystal was presented in our previous paper.^13^ The grown crystal pieces were ground in an agate mortar and turned into powder, and the structural and thermal properties of these powder materials were investigated in the presented paper. Structural characterization of the BGO crystals was conducted via X-ray diffraction (XRD) analysis using a Bruker D8 Advance X-ray Diffractometer over a diffraction angle range of 20–90 deg with a scanning at a rate of 0.04°/min. A monochromatic Cu Kα beam (λ = 1.54056 Å) served as the X-ray source, with patterns recorded at 40 kV and 40 mA. Energy-dispersive spectroscopy (EDS) and scanning electron microscopy (SEM) measurements were performed to determine the atomic composition of the constituent elements and surface morphology using a Hitachi SU8700 SEM (HV of 20 kV, dwell time of 1 μs, beam intensity of 10 nA, spot size 30 nm, and WD 10 mm) equipped with an energy-dispersive X-ray spectrometer. The morphological analysis was also performed using a Jeol 2100F 200 kV R­(TEM) transmission electron microscope. TGA measurements were performed on a Hitachi STA7300 device at a heating rate of 10 K/min between 303 and 1223 K. Differential scanning calorimetry (DSC) experiments were performed, utilizing the HITACHI DSC 7020 thermal analysis unit. Tests were executed within a temperature range of 600–635 K under an argon atmosphere, employing heating at heating rates of 15, 20, and 25 K/min. Each test utilized specimens weighing 10 mg enclosed in aluminum pans.

## 3 Results and Discussion

### 3.1 Structural Properties

XRD measurements were carried out to reveal the crystalline properties of the Bi_12_GeO_20_ compound grown by the Czochralski method. For XRD measurements, Bi_12_GeO_20_ crystals were ground into powder by pounding in a mortar. In bulk crystals, the crystal surface orientation will be limited and therefore will not give many peaks. When the bulk crystal is ground into powder, the orientation of each powder will be random. The powder XRD pattern shows many diffraction peaks typical of powder samples due to the random orientation of crystallites. Each crystallographic plane contributes to the diffraction pattern and results in peaks corresponding to the cubic symmetry of Bi_12_GeO_20_. The experimental XRD pattern obtained as a result of measurements made on the powder sample in the range 20–90 ° is shown in Figure 2. The presence of a very large number of observed sharp and well-defined peaks in the XRD pattern shows that the crystal growth process has taken place successfully. The XRD data of the BGO crystal (see S1 Data in Supporting Information) were analyzed using the MAUD program, considering the standard peak (CIF File: 1534137 ^20^), and the lattice parameter was determined to be 1.01524 nm. The fitting process, as shown in Figure 2, was carried out successfully, with the calculated pattern (red line) closely matching the experimental data (black circles). The data we prepared regarding XRD peaks according to the specified standard card are shown in Table 1. The peaks of the Bi_12_GeO_20_ compound in the XRD pattern are also consistent with the peaks given in the previously reported experimentally XRD patterns.^18,21^ Table 2 summarizes the key parameters obtained from the Rietveld refinement analysis. The *R*_wp_ value (R-weighted pattern), calculated as 19.1%, indicates a reasonable fit between the experimental and the calculated patterns. The *R*_b_ value of 14.8% reflects an acceptable agreement between the observed and calculated peak intensities. While the sig (χ^2^) value of 4.18 suggests that the fit is satisfactory, it also highlights areas for potential refinement. The *R*_exp_ value of 4.6% represents the expected statistical error, suggesting that further optimization, such as refining background coefficients and instrumental parameters, could improve the fit quality. Table 3 presents the atomic positions for the Bi_12_GeO_20_ structure obtained from the Rietveld refinement analysis using the MAUD program. The table includes the atom labels, types, and fractional coordinates (*x*, *y*, *z*) within the unit cell, which describe the precise locations of atoms in the crystal lattice. These refined positions align well with the expected crystallographic model, supporting the reliability of the structural parameters determined in this study.

**Figure 2:**
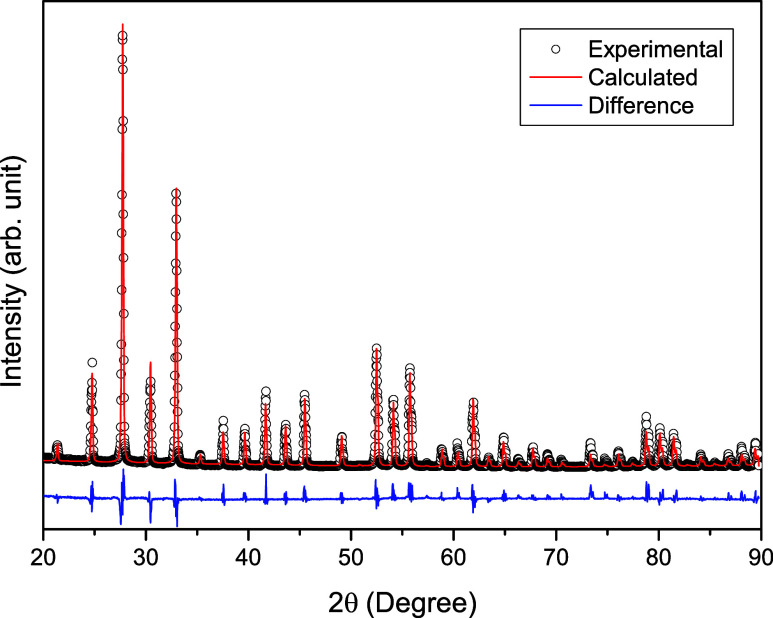
Rietveld refinement of the XRD pattern of the Bi_12_GeO_20_ crystal.

**Table 1:** XRD Data for the Bi_12_GeO_20_ Crystal

2θ	*hkl*	*I*/*I*_0_	2θ	*hkl*	*I*/*I*_0_
21.38	211	5.05	63.42	444	2.12
24.78	220	24.15	64.88	345	6.81
27.75	310	100	66.29	046	1.89
30.47	222	19.80	67.77	721	455
32.95	321	63.38	69.15	246	2.93
35.29	400	2.80	70.51	037	1.66
37.56	330	10.68	73.34	651	5.54
39.66	024	8.83	74.74	800	1.92
41.71	332	17.49	76.08	147	3.58
43.64	422	9.72	77.46	820	1.20
45.49	134	16.69	78.80	356	11.63
49.11	521	6.96	80.13	660	8.93
52.50	035	27.48	81.45	138	7.68
54.15	600	15.58	84.11	257	2.83
55.75	532	22.89	85.47	840	0.99
58.89	541	3.95	86.81	910	2.93
60.37	622	5.44	88.09	842	4.65
61.94	631	14.89	86.42	921	5.59

**Table 2:** Crystal Structure Properties and Figure-of-Merit (FoM) Values of Refinement Using MAUD Software

crystal structure	space group	lattice parameter (nm)	cell volume (nm^3^)	crystalline size (nm)	microstrain
	*I*2̅ 3̅	*a* = 1.0155(4)	1.0473(5)	38.6	–6.4 × 10^–4^

FoM values	sig (χ^2^)	*R*_w_ (%)	*R*_wp_ (%)	*R*_b_ (%)	*R*_exp_ (%)
	4.18	19.1	25.0	14.8	4.6

**Table 3:** Atomic Positions for the Bi_12_GeO_20_ Structure

atom label	atom type	fractional *x*	fractional *y*	fractional *z*
O3	O^2–^	0.168426(6)	0.168426(6)	0.168426(6)
O2	O^2–^	0.780574(1)	0.780574(1)	0.780574(1)
Bi1	Bi^3+^	0.823440(9)	0.684304(4)	0.983566(2)
Ge1	Ge^4+^	0	0	0
O1	O^2–^	0.870986(8)	0.70784(0)	0.516732(9)

Williamson and Hall postulated that the broadening observed in diffraction lines arises from contributions related to both the size of the crystallites and the presence of strain within the crystal lattice. The Williamson–Hall equation is expressed as follows^22^

1 $$\beta_{\mathrm{hkl}}=\beta_{d}+\beta_{\varepsilon }$$

where β_*hkl*_ represents the full width at half-maximum (fwhm) of the diffraction peak corrected for instrumental broadening, β_*d*_ denotes the broadening attributable to the size of the crystallites, and β_*ε*_ signifies the broadening originating from strain-induced effects. The crystallite size broadening and strain-induced broadening are calculated using the following expressions

2 $$\beta_{d}=0.94\lambda /D\cos\theta$$

3 $$\beta_{\varepsilon }=4\varepsilon \tan\theta$$

where *D* is the average crystallite size, λ = 0.154060 nm is the X-ray wavelength, and *ε* is the microstrain. Hence, the broadening of the diffraction line encompasses both crystallite size and strain contributions, as described by the equation^22^

4 $$\beta_{\mathrm{hkl}}=\beta_{d}+\beta_{\varepsilon }=\frac{0.94\lambda }{D\cos\theta }+4\varepsilon \tan\theta$$

Based on this equation, plotting βcos θ against 4sin θ yields a slope representing the strain and y-intercept equivalent to *kλ*/D. Figure 3 displays the experimental data (depicted as circles) along with the linear fit (illustrated by the solid line). The determined values for strain and crystalline size were −6.4 × 10^–4^ and 38.6 nm, respectively. The negative slope observed in Figure 3 suggests that microstrains are not the primary source of broadening. Based on these findings, it can be deduced that the impact of the domain/crystallite size and microstrains is insignificant. Dislocation density denotes the abundance or density of dislocations present within a material. Dislocations, which are structural imperfections within the crystal lattice of a material, emerge when atoms deviate from their optimal positions within the lattice structure. These imperfections exert a substantial influence on the mechanical characteristics of materials. Dislocation density (δ) is expressed in terms of the crystallite size as follows

5 $$\delta =\frac{1}{D^{2}}$$

**Figure 3:**
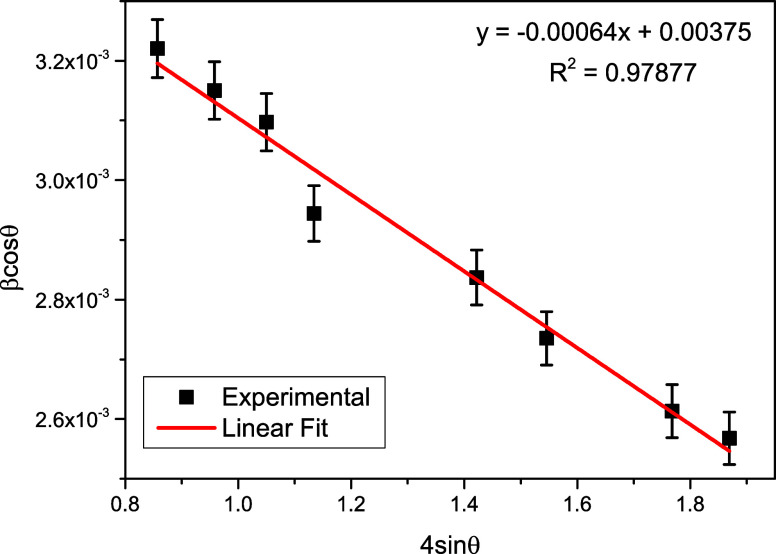
Williamson–Hall plot of βcosθ against 4sinθ. Solid lines indicate the linear fit.

The dislocation density of the Bi_12_GeO_20_ crystal was calculated as 6.7 × 10^14^ m^–2^ using eq 5.

The elemental composition and spatial distribution of the Bi_12_GeO_20_ crystal were investigated by using EDS and SEM mapping analysis. Figure 4a,b illustrates the SEM micrographs of the crystal. The EDS spectra, acquired from two distinct points on the crystal, are presented in Figure 4c,d, providing insights into the composition and weight ratios of the constituent elements within the compound. Although the order of the weight ratios obtained as a result of the analysis according to the elements is as expected: (*W*_Bi_ > *W*_O_ > *W*_Ge_), some differences are noticeable according to the measurement results taken from different points. The EDS method has limitations in the detection of light elements (e.g., oxygen) and cannot provide sufficient reliability in terms of the accuracy of oxygen content. Especially in the presence of heavy elements such as Bi, light elements may be overshadowed in the signal. Therefore, the oxygen content results obtained by EDS only provide an estimated value. While SEM measurements show that the observed particles are micrometer-sized, the crystallite size obtained from XRD analysis was determined to be approximately 48.4 nm. This difference is due to the fact that XRD determines the size of each single crystallite, while SEM shows larger particles formed by multiple crystallites coming together. This is a difference that is frequently encountered, especially in micro- and nanosized materials.

**Figure 4:**
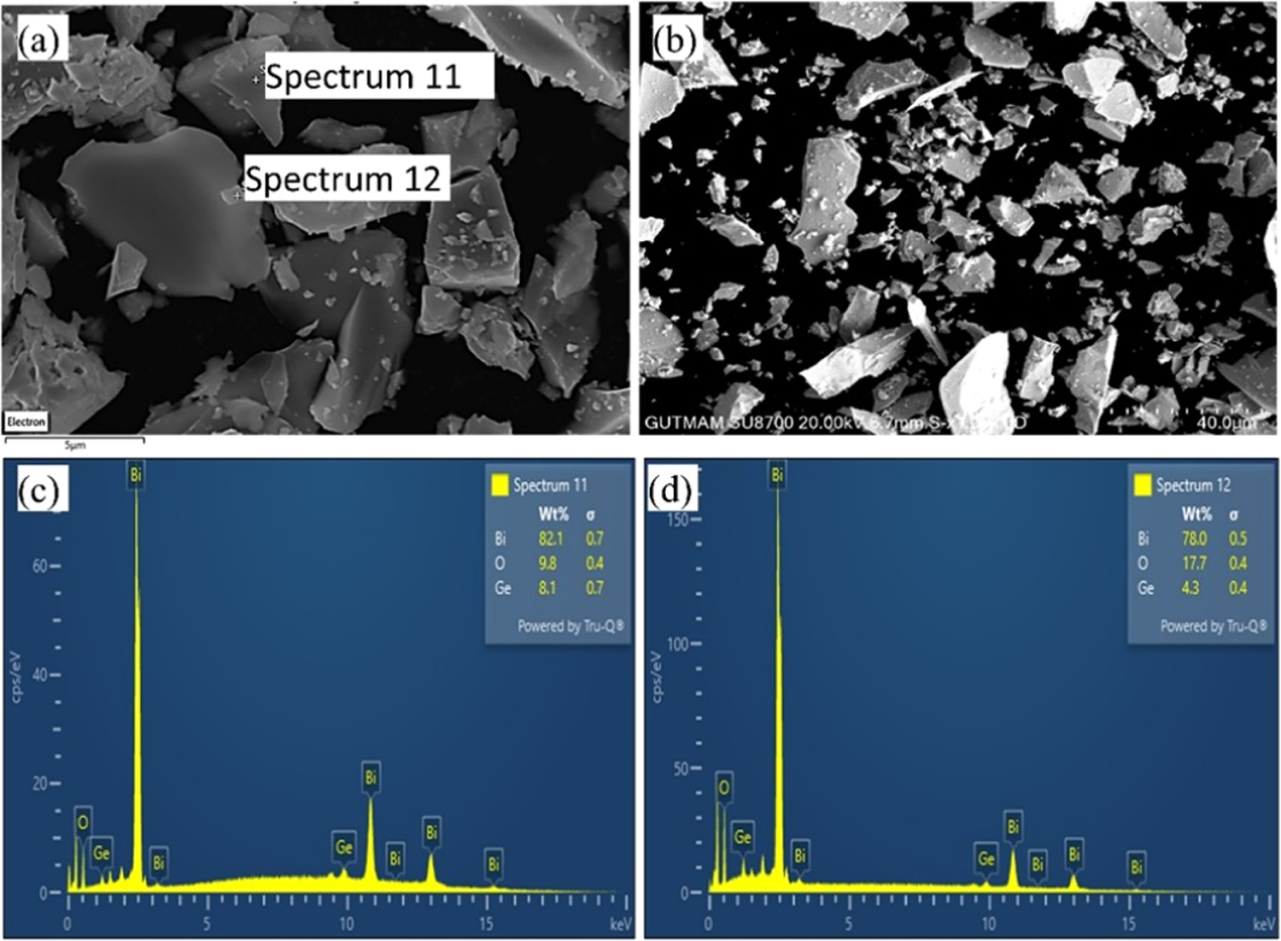
Panels (a, b) are SEM images of the Bi_12_GeO_20_ crystal. (c, d) Corresponding EDS spectra.

The EDS line scanning profile of the Bi_12_GeO_20_ crystal is represented in Figure 5. As can be seen from the figure, the behavior of Bi, Ge, and O elements is similar depending on the position. While the Bi element has the highest intensity, as expected, the intensity behaviors of the other two elements, Ge and O, are seen close to each other. Moreover, the elemental mapping of the grown crystal is depicted in Figure 6, which supports the homogeneous distribution of the corresponding Bi, Ge, and O elements. Although there are some minor compositional differences depending on the location in the EDS analyses, it can be concluded from these data that the Bi_12_GeO_20_ crystal was grown successfully. TEM image in Figure 7 reveals the morphological properties of the Bi_12_GeO_20_ crystal. In the image, distinct and sharp morphological features on a scale of approximately 100 nm are observed. In addition, the selective area electron diffraction (SAED) pattern on the screen confirms that the crystal has a highly ordered and uniform crystal structure. The regular dot arrays in the SAED pattern indicate that the material has a specific crystal phase and symmetry, providing important information about the crystallographic orientations of the Bi_12_GeO_20_ crystal.

**Figure 5:**
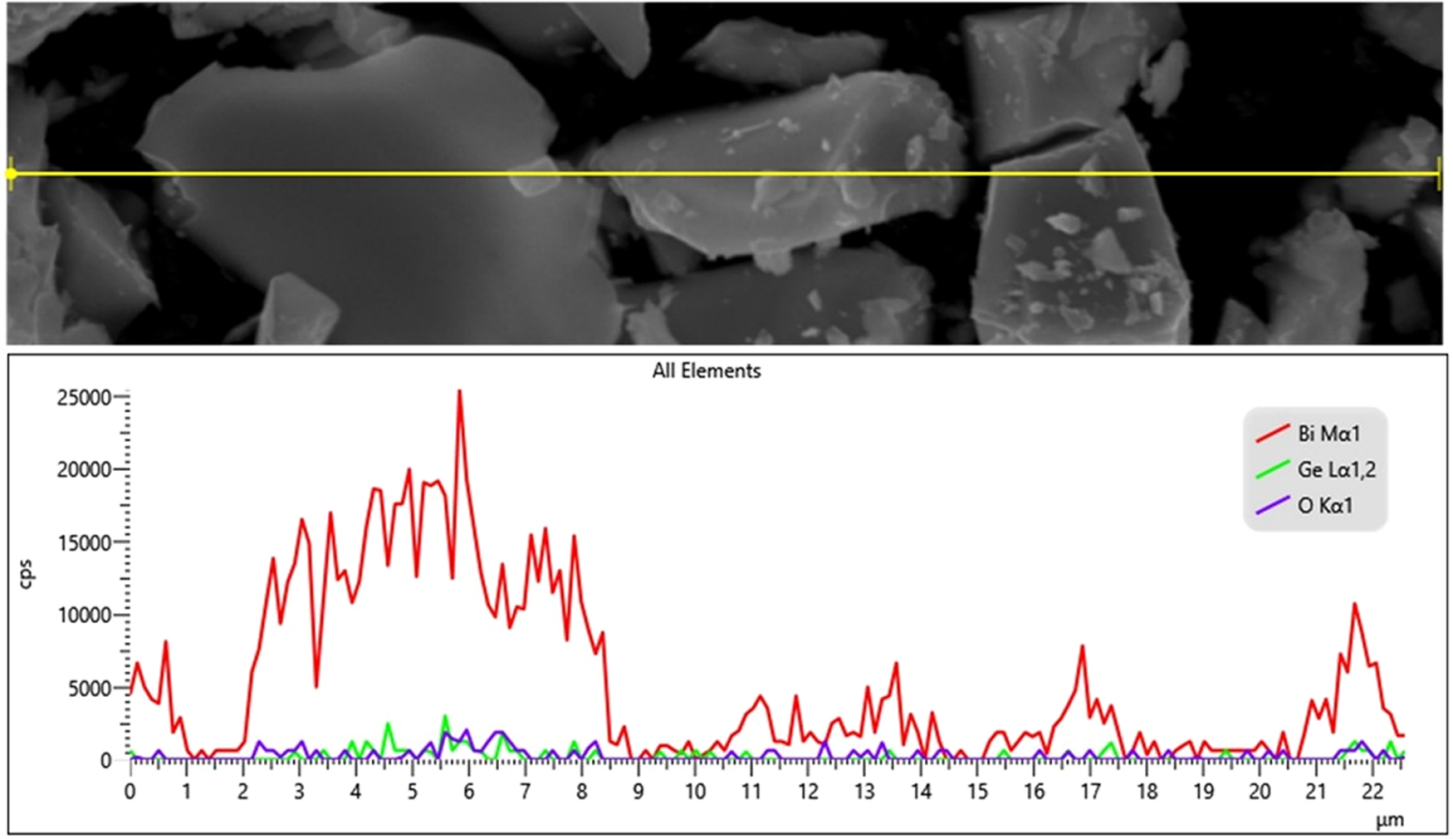
EDS line analysis of the Bi_12_GeO_20_ crystal.

**Figure 6:**
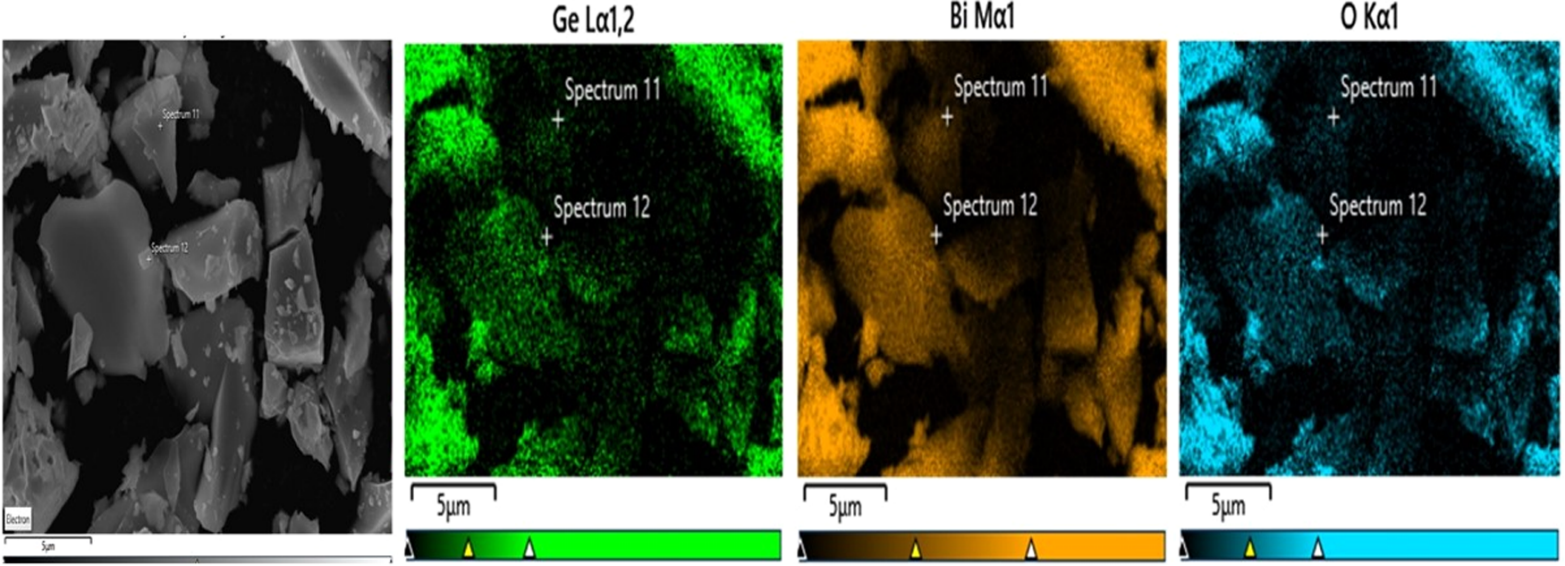
Elemental mapping of Bi, Ge, and O elements.

**Figure 7:**
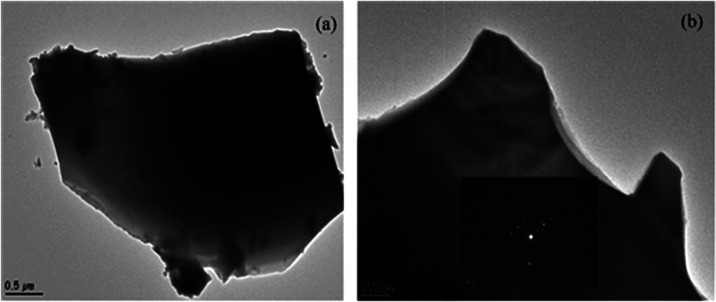
TEM images at (a) low magnification and (b) corresponding SAED pattern.

### 3.2 Thermal Properties

Thermogravimetry (TGA) analysis reveals the change in the weight of materials depending on the temperature and provides important information about the thermal stability of the materials. Knowing the weight change that occurs in the material at different temperatures is necessary to select suitable and temperature-resistant materials for certain applications. Figure 8 indicates the TGA thermograms of the Bi_12_GeO_20_ crystal in the 303–1223 K range, and TGA data are presented in the Supporting Information (see S2 Data in Supporting Information). As can be seen from the figure, no significant weight loss was observed in the Bi_12_GeO_20_ crystal up to ∼863 K. This indicates that the BGO crystal is stable in the temperature region up to 863 K. After 863 K, weight loss started because of the heating crystal. As seen from the weight loss vs temperature graph, the decreases exhibited different behaviors in the regions 863–1048 K, 1048–1193 K, and above 1193 K. This indicates that the first decomposition occurred in the region between 863 and 1048 K and the second decomposition took place between 1048 and 1193 K. While the weight loss was approximately 14% in the first decomposition region, a 12% loss was observed in the second decomposition region. In the region above 1193 K, a serious change was observed in the weight loss per unit temperature. Although a full interpretation cannot be made for this region according to the available experimental data, it can be said that melting occurs in the region above 1193 K, considering the reported melting temperature of 1196 K for the BGO crystal.^23^

**Figure 8:**
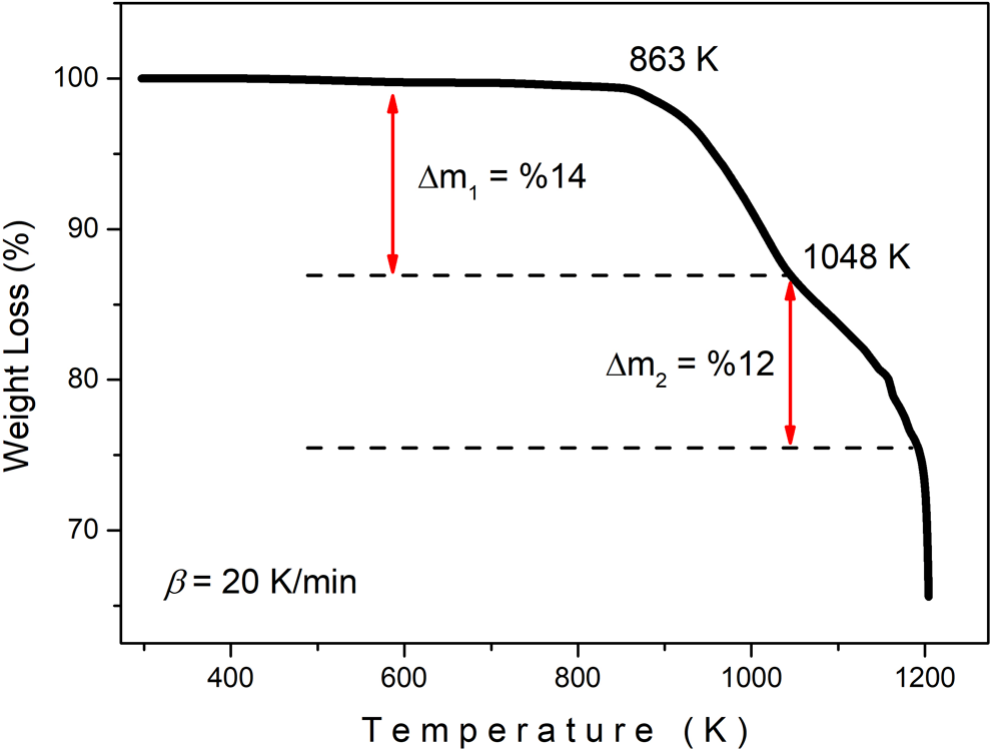
TGA curve of the Bi_12_GeO_20_ crystal.

Considering both decompositions as first-order reactions, activation energies of the associated decomposition processes were determined by applying the following Coats–Redfern expression^24,25^

6 $$\ln\left[\frac{-\ln\left(1-\alpha \right)}{T^{2}}\right]=\ln\left(\frac{\mathrm{AR}}{\beta E_{a}}\right)-\frac{E_{a}}{\mathrm{RT}}$$

where *A* is the frequency factor, *R* = 8.314 J/mol.K is the gas constant, β = 20 K/min is the heating rate, *E*_a_ is the activation energy, *T* is the temperature, and α is the conversion defined as^25^

7 $$\alpha =\frac{m_{0}-m_{t}}{m_{0}-m_{f}}$$

where *m_0_*, *m_t_*, and *m_f_* symbolize the initial mass, mass at time *t*, and final mass of the compounds, respectively. According to eq 6, the slope of the ln­[−ln­(1−α)/*T* ^2^] vs 1/*T* plot is equal to −*E*_*t*_/*R*. Figure 9 shows the linear fit analyses performed for both decomposition phases and the activation energies obtained using the slope found as a result of the linear fit. While the activation energy for the first decomposition phase occurring in the temperature range of 863–1048 K was found to be 99.7 kJ/mol, the activation energy for the second decomposition phase occurring between 1048 and 1193 K was found to be 33.1 kJ/mol (Table 4).

**Figure 9:**
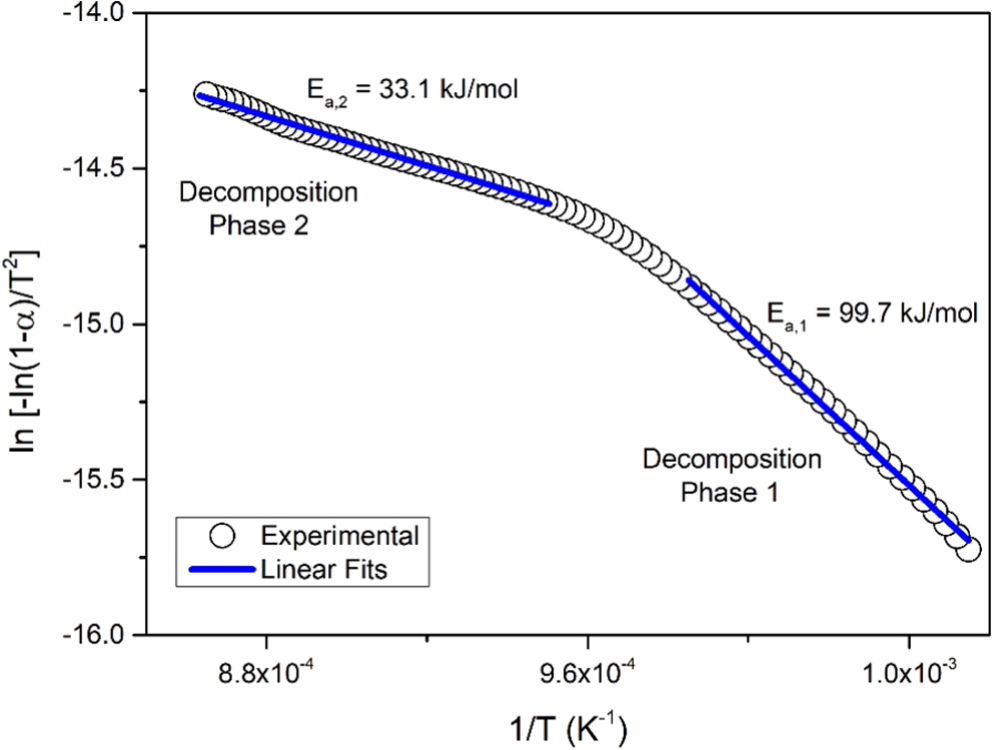
*ln*[−ln­(1−α)/*T* ^2^] vs 1/*T* plot.

**Table 4:** Kinetic Parameters for the Decomposition of Bi_12_GeO_20_

decomposition stage	*E* (kJ/mol)	*A* (min^–1^)	*R* ^2^
1	99.7	1.2 × 10^4^	0.9983
2	33.1	1.6	0.9956

At this point, it will be valuable to compare the TGA results of the Bi_12_GeO_20_ crystal with those of materials with similar structures. In comparison with Bi_12_SiO_20_, which has a decomposition activation energy of 199 kJ/mol in the 1023–1192 K range, Bi_12_GeO_20_ exhibits much lower activation energies of 99.7 and 33.1 kJ/mol in its respective decomposition regions (863–1048 and 1048–1193 K).^26^ This suggests that Bi_12_GeO_20_ has lower thermal stability relative to Bi_12_SiO_20_, as its decomposition process occurs at lower energy thresholds. The thermal stability of Bi_12_GeO_20_ is notably higher than that of Bi_12_MnO_20_ nanopowders, which begins decomposing at around 373 K and exhibits a substantial 23.7% mass loss by 1173 K.^27^ In contrast, Bi_12_GeO_20_ starts to experience significant mass losses only at 863 K, indicating a more stable thermal profile up to high temperatures. Compared to Bi_12_TiO_20_, which exhibits continuous weight loss from 298 to 873 K, Bi_12_GeO_20_ shows greater thermal stability by undergoing significant weight changes at temperatures above 863 K.^28^ This feature could make Bi_12_GeO_20_ more suitable for high-temperature applications requiring minimal decomposition.

Figure 10 shows the experimental plots of DSC measurements performed on the BGO crystal at different heating rates, and DSC data are presented as Supporting Information (see S3 Data in Supporting Information). The heating rates of 15, 20, and 25 K/min were selected based on experimental observations: heating rates below 15 K/min resulted in poorly defined peaks, while rates above 25 K/min could introduce temperature lag issues. Furthermore, smaller intervals than 5 K/min were avoided due to minimal temperature shifts, which could increase the margin of error in the analysis. It has been observed that the peak minimum points shift to higher temperatures by increasing the heating rate. Peak minimum points are recorded as 618.4, 620.4, and 621.6 K for heating rates of 15, 20, and 25 K/min, respectively. The main reasons why peaks shift to higher values as the heating rate increases are as follows:^29,30^ (i) *thermal lag*: thermal lag occurs when higher heating rates limit the sample’s ability to equilibrate with the temperature increase, creating a delay between the sample and its surroundings. Consequently, the sample needs higher temperatures to achieve the equivalent thermal energy level observed at lower heating rates, resulting in a shift of observed peaks toward higher temperatures. (ii) *Kinetic effects*: kinetic effects arise from the rearrangement of molecular or atomic structures during phase transitions. With higher heating rates, the speed of these structural changes increases, driven by the rapid temperature increase. Consequently, phase transitions take place at elevated temperatures to surpass the activation energy barriers linked to these transitions. (iii) *Heat transfer effects*: heat transfer effects become prominent with higher heating rates, potentially constraining the speed at which heat is transferred to the sample. This limitation can induce temperature variations within the sample, impacting the observed peak temperatures and resulting in an upward shift.

**Figure 10:**
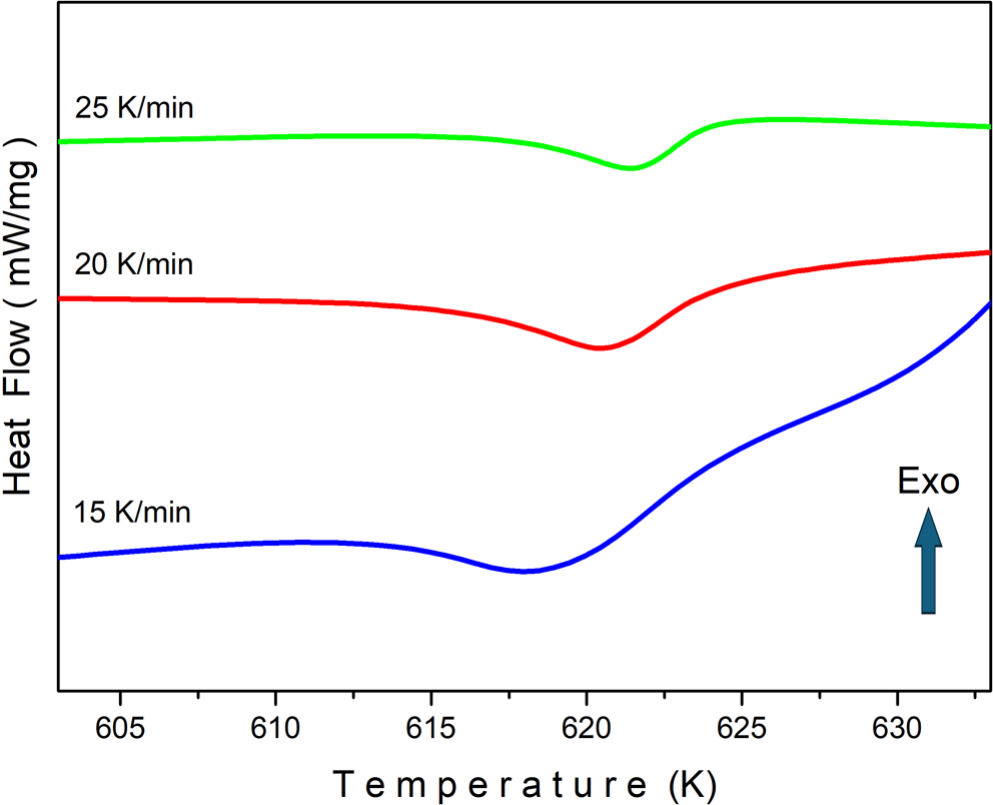
DSC plots of the Bi_12_GeO_20_ crystal for three different heating rates.

The endothermic peak observed in the DSC measurements around 618 K suggests a potential association with thermal decomposition processes. Several potential processes could contribute to this peak such as phase transitions, crystallization, water evaporation, or structural changes due to defects. However, based on the available literature, no phase transition in the BGO crystal has been reported so far. Additionally, the high-quality nature of the crystal and the low likelihood of the crystal containing water molecules suggest that the most probable explanation for this endothermic behavior is thermal decomposition. Bi_2_O_3_ is a major constituent of the BGO compound, and it is highly plausible that, upon thermal decomposition, Bi_2_O_3_ would form. GeO_2_ is another likely product of the decomposition reaction. This is supported by the following chemical equation

$$2{\mathrm{Bi}}_{12}\mathrm{Ge}O_{20}\to 12{\mathrm{Bi}}_{2}O_{3}+2{\mathrm{GeO}}_{2}$$

The second weight loss region observed in the TGA graph (1048–1193 K) is likely associated with the decomposition of Bi_2_O_3_ and/or GeO_2_ compounds, which may release oxygen (O_2_) during the process. The fact that no significant weight loss is observed up to 863 K further supports this interpretation. In the literature, it has been reported that GeO_2_ decomposes to GeO and O_2_ in the temperature range of 673–773 K.^27^ The corresponding chemical reaction is as follows

$$2\mathrm{Ge}O_{2}\to 2\mathrm{GeO}+O_{2}$$

Combining the TGA and DSC data, we hypothesize that around 618 K, the BGO crystal undergoes thermal decomposition, likely to form GeO_2_ and O_2_. In the DSC analysis of Bi_12_SiO_20_, an endothermic peak was also observed at approximately 621 K, which is close to the peak detected at 618 K in Bi_12_GeO_20_.^25^ This similarity may indicate a comparable thermal event, potentially related to the same type of thermal decomposition, in these two sillenite crystals. The mass losses observed at temperatures above 863 K are primarily attributed to the evaporation of GeO_2_, GeO, O_2_, and possibly Bi_2_O_3_. This explanation aligns with both the thermal behavior observed in TGA and the endothermic peak seen in the DSC measurements, suggesting that these processes are interconnected and crucial for understanding the thermal stability and decomposition mechanisms of BGO. The activation energies calculated by the Coats–Redfern method reveal the thermal rates of the two different decomposition stages and the kinetic properties of these processes. The activation energy of the first process, 99.7 kJ/mol, reflects the high energy requirement of the first reaction at the decomposition initiation temperature. This high activation energy indicates a significant energy barrier for the dissolution of bonds in the internal structure of the crystal and the dissociation of the compounds. This indicates that the stability of the BGO crystal is high and that it has a significant energy level for thermal dissociation. The activation energy of 33.1 kJ/mol calculated in the second process indicates that the chemical reactions in this process occur with lower energy, and therefore, a faster dissociation process is present. This lower energy generally means that less bond dissociation and a simpler reaction mechanism may be present. In addition, this value supports the occurrence of less complex dissociation reactions such as the decomposition of GeO_2_.

The following Kissenger equation was used to calculate the activation energy associated with the observed endothermic process in the DSC plot^31^

8 $$\ln\left(\beta /T_{m}^{2}\right)=-\left(E_{a}/RT_{m}\right)+C$$

where *T*_m_ and *C* represent the peak minimum temperature and temperature-independent constant, respectively. According to this equation, *ln*(β/*T*_m_^2^) vs 1/*T*_m_ graph exhibits a linear behavior, and the slope of this linearity is equal to −*E*_a_/*R*. The graph mentioned and the applied linear fit line are shown in Figure 11. Using the slope of this linear fitted line, the activation energy was calculated as 495 kJ/mol.

**Figure 11:**
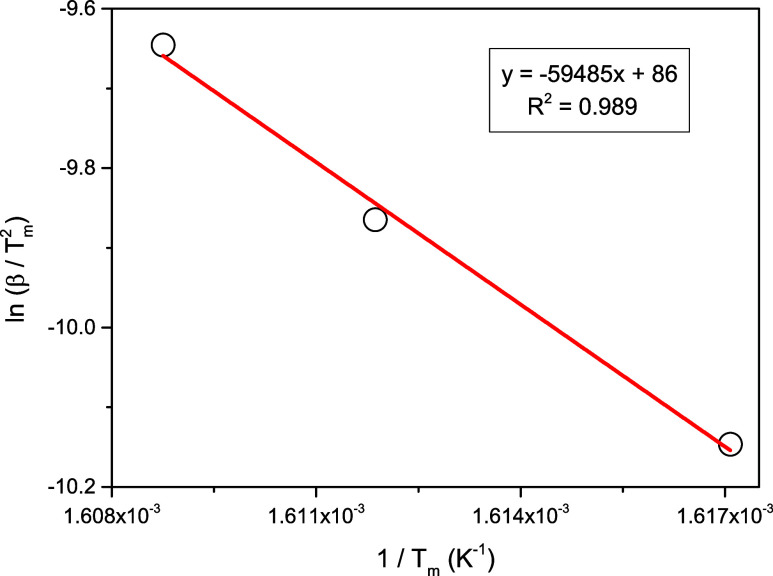
ln­(β/*T*_m_^2^) vs 1/*T*_m_ plot for the Kissenger analysis.

## 4 Conclusions

In this study, the Bi_12_GeO_20_ (BGO) crystal was comprehensively characterized in terms of its structural and thermal properties. The XRD analysis confirmed its cubic crystal structure with a lattice parameter of 1.01554 nm. The microstrain, crystal size, and dislocation density obtained from the Rietveld refining were calculated as −6.4 × 10^–4^, 38.6 nm, and 6.7 × 10^14^ m^–2^, respectively. SEM and EDS mapping demonstrated a uniform elemental distribution, further supporting the homogeneity of the grown crystal. Thermal analysis revealed that the crystal remained structurally stable up to 863 K, exhibiting no significant mass loss in this range, which underscores its thermal robustness. However, decomposition processes were initiated above this temperature, as evidenced by notable weight losses in TGA and corresponding thermal events in DSC. The weight loss vs temperature graph obtained as a result of TGA measurements showed that the crystal experienced serious weight losses in the regions of 863–1048 and 1048–1193 K. Weight losses in the range of 863–1048 and 1048–1193 K were associated with decomposition processes. The activation energies of these processes were found to be 99.7 and 33.1 kJ/mol by using the Coats–Redfern equation. The heat flow vs temperature graph obtained from DSC measurements revealed the existence of an endothermic process around 618 K. As a result of analyzing the experimental data obtained using different heating rates with the Kissenger method, the activation energy of the relevant process was found to be 495 kJ/mol. Such decomposition leads to the formation of secondary phases like Bi_2_O_3_ and GeO_2_, which can adversely affect the thermal and electrical behavior of the crystal. Overall, the high thermal stability of BGO below 863 K indicates its suitability for applications in thermally demanding environments including high-temperature sensors and energy storage systems. Nonetheless, care must be taken when considering its use at higher temperatures, where degradation could compromise its performance in electronic or optoelectronic devices.

## Supplementary Material



## Data Availability

The data underlying this study are available in the published article and its Supporting Information.

## Notes

• **S1 Data**: XRD; **S2 Data**: TGA; and **S3 Data**: DSC (XLSX)
